# Lysosomal phospholipase A2 contributes to the biosynthesis of the atypical late endosome lipid bis(monoacylglycero)phosphate

**DOI:** 10.1038/s42003-023-04573-z

**Published:** 2023-02-23

**Authors:** Jacinda Chen, Amaury Cazenave-Gassiot, Yimeng Xu, Paola Piroli, Robert Hwang, Laura DeFreitas, Robin Barry Chan, Gilbert Di Paolo, Renu Nandakumar, Markus R. Wenk, Catherine Marquer

**Affiliations:** 1 Taub Institute for Research on Alzheimer’s Disease and the Aging Brain, Columbia University Irving Medical Center, New York City, NY 10032 USA; 2 Department of Biochemistry and Precision Medicine Translational Research Program, Yong Loo Lin School of Medicine, National University of Singapore, Singapore, 117597 Singapore; 3 Singapore Lipidomics Incubator, Life Sciences Institute, National University of Singapore, Singapore, 117456 Singapore; 4 Biomarkers Core Laboratory, Irving Institute for Clinical and Translational Research, Columbia University Irving Medical Center, New York City, NY 10032 USA; 5 Department of Pathology and Cell Biology, Columbia University Irving Medical Center, New York City, NY 10032 USA; 6 Present Address: AliveX Biotech, Shanghai, China; 7 Present Address: Denali Therapeutics Inc., South San Francisco, CA USA

**Keywords:** Lysosomes, Lipid-storage diseases

## Abstract

The late endosome/lysosome (LE/Lys) lipid bis(monoacylglycero)phosphate (BMP) plays major roles in cargo sorting and degradation, regulation of cholesterol and intercellular communication and has been linked to viral infection and neurodegeneration. Although BMP was initially described over fifty years ago, the enzymes regulating its synthesis remain unknown. The first step in the BMP biosynthetic pathway is the conversion of phosphatidylglycerol (PG) into lysophosphatidylglycerol (LPG) by a phospholipase A2 (PLA2) enzyme. Here we report that this enzyme is lysosomal PLA2 (LPLA2). We show that LPLA2 is sufficient to convert PG into LPG in vitro. We show that modulating LPLA2 levels regulates BMP levels in HeLa cells, and affects downstream pathways such as LE/Lys morphology and cholesterol levels. Finally, we show that in a model of Niemann-Pick disease type C, overexpressing LPLA2 alleviates the LE/Lys cholesterol accumulation phenotype. Altogether, we shed new light on BMP biosynthesis and contribute tools to regulate BMP-dependent pathways.

## Abstract (web summary)

Lysosomal phospholipase A2 (LPLA2) is the PG to LPG converting enzyme in the BMP biosynthetic pathway, and changes in LPLA2 levels in cells affect cholesterol levels and late endosome/lysosome morphology.

## Introduction

Select lipids have emerged as key regulators of intracellular trafficking and consequently, as essential components of cellular homeostasis. Amongst them, bis(monoacylglycero)phosphate (BMP, also called lysobisphosphatidic acid or LBPA) is uniquely localized to late endosomes/lysosomes (LE/Lys), where it is further concentrated in intralumenal vesicles (ILVs)^1^. LE/Lys are a sorting crossroad for both proteins and lipids that will be targeted towards retrograde transport, degradation or extracellular export. Indeed, in addition to their essential intracellular sorting function, LE/Lys can also fuse with the plasma membrane and release ILVs as nanovesicles in the extracellular milieu, known as exosomes.

As BMP presents with the intrinsic capacity to induce the formation of multivesicular liposomes in vitro, BMP and its binding partner Alix are considered to be key players in the formation of ILVs^2,3^. BMP also affects the correct sorting and degradation of proteins and lipids. For example, it plays a key role in the retrograde transport of mannose-6-phosphate receptor to the trans-Golgi network^1^ and facilitates the correct degradation of sphingolipids^4^. BMP also promotes cell survival through the stabilization of lysosomes^5^.

Importantly, BMP controls cholesterol storage capacity in LE/Lys and subsequently, intracellular cholesterol levels^6,7^. The majority of cellular cholesterol originates from extracellular cholesteryl ester-rich low-density lipoprotein (LDL) particles that are internalized by the LDL receptor and trafficked to the lumen of LE/Lys. Cholesteryl esters are then hydrolyzed by lysosomal acid lipase into free cholesterol. Free cholesterol can then be incorporated into BMP-rich ILVs membranes before being redistributed to the LE/Lys limiting membrane and then to the rest of the cell^7^. It was recently proposed that Niemann Pick type C 2 (NPC2) plays a key role in bringing together cholesterol and BMP-rich ILVs membranes with the limiting membrane of LE/Lys, as it can bind both BMP and proteins on the LE/Lys limiting membrane (e.g., NPC1, LAMP1)^8^.

Unsurprisingly, given the number of essential homeostasis pathways it regulates, BMP has also been implicated in a vast number of diseases. BMP is required for the initial entry step of specific viruses—e.g., vesicular stomatitis virus, dengue virus, Lassa virus—through the back-fusion of ILVs with the limiting membrane of LE/Lys and release of the virus nucleocapsid within the cell cytoplasm^9–12^. Moreover, most neurodegenerative diseases show alterations in BMP-regulated pathways and BMP levels are profoundly altered in patients with neurodegenerative disorders of childhood and aging, as well as in model systems of these diseases; e.g., lysosomal storage disorders^13–16^, Alzheimer’s disease^17,18^, Down Syndrome^17^ and dementia with Lewy bodies^19^. Finally, the presence and abundance of BMP regulate the intracellular delivery of certain candidate drugs, such as cationic cell-penetrating peptides^20^ and antisense oligonucleotides^21^.

Modulating endogenous BMP levels would thus be of crucial interest not only to understand the molecular mechanisms involved in the pathogenesis of these disorders, but also potentially as a therapeutic tool. These studies are limited by the fact that although BMP has been described as early as 1967^22^, and despite the fact that its levels and localization can be probed by a specific antibody^1^, its exact biosynthetic enzymes remain unknown. Here we propose a major advance in answering this question lingering for over 55 years.

It has been established that phosphatidylglycerol (PG) is the precursor in the de novo synthesis of BMP^23^. In the currently prevailing model of BMP biosynthesis^24^, the first and limiting step involves the conversion of sn-3:sn-1’ PG to sn-3:sn-1’ LPG by an enzyme with phospholipase A2 (PLA2) activity. The sn-3:sn-1’ LPG intermediate then goes through two transacylation steps to give the final product, sn-1:sn-1’ BMP. Of all sixteen classes of PLA2 enzymes, only lysosomal PLA2 (LPLA2 or Group XV LPLA2), coded by *PLA2G15*, localizes to the LE/Lys^25^. We thus tested whether LPLA2 could be the PLA2 enzyme regulating the first and limiting step of the BMP biosynthetic pathway. LPLA2 was initially identified in Madin-Darby Canine Kidney cells^26^ and then purified from bovine brain^27^. The human and mouse isoforms contain 412 amino acids, with a signal peptide cleavage site, a lipase AXSXG motif, and four N-linked glycosylation sites^28^. A serine, aspartic acid, and histidine catalytic triad is essential for enzymatic activity^29^. LPLA2 displays 1-O-acylceramide synthase activity as well as PLA1 and PLA2 activities, is Ca^2+^-independent and has an acidic pH optimum^30^. Its known substrates include phosphatidylcholine (PC), phosphatidylethanolamine (PE), PG, and phosphatidylserine (PS) but not phosphatidylinositol (PI) or sphingomyelin (SM)^31^.

In this study, we first confirm that LPLA2 is sufficient to catalyze the conversion of PG to LPG in an in vitro liposomes assay. We further show that over-expressing or down-regulating LPLA2 leads to increased or decreased BMP levels in human cell lines, respectively. In addition, we show that decreasing LPLA2 levels also affect BMP-dependent pathways, such as LE/Lys homeostasis and cholesterol regulation. Finally, we demonstrate that in a cellular model of Niemann-Pick disease type C, a lysosomal storage disorder in which cholesterol accumulates in LE/Lys, overexpressing LPLA2 alleviates the cholesterol accumulation phenotype. Taken together, our results strongly support that LPLA2 plays a key functional role in the BMP biosynthetic pathway and provide tools to regulate BMP levels and BMP-dependent pathways in control and diseased model systems.

## Results

### LPLA2 catalyzes the conversion of PG to LPG in vitro

**Fig. 1:**
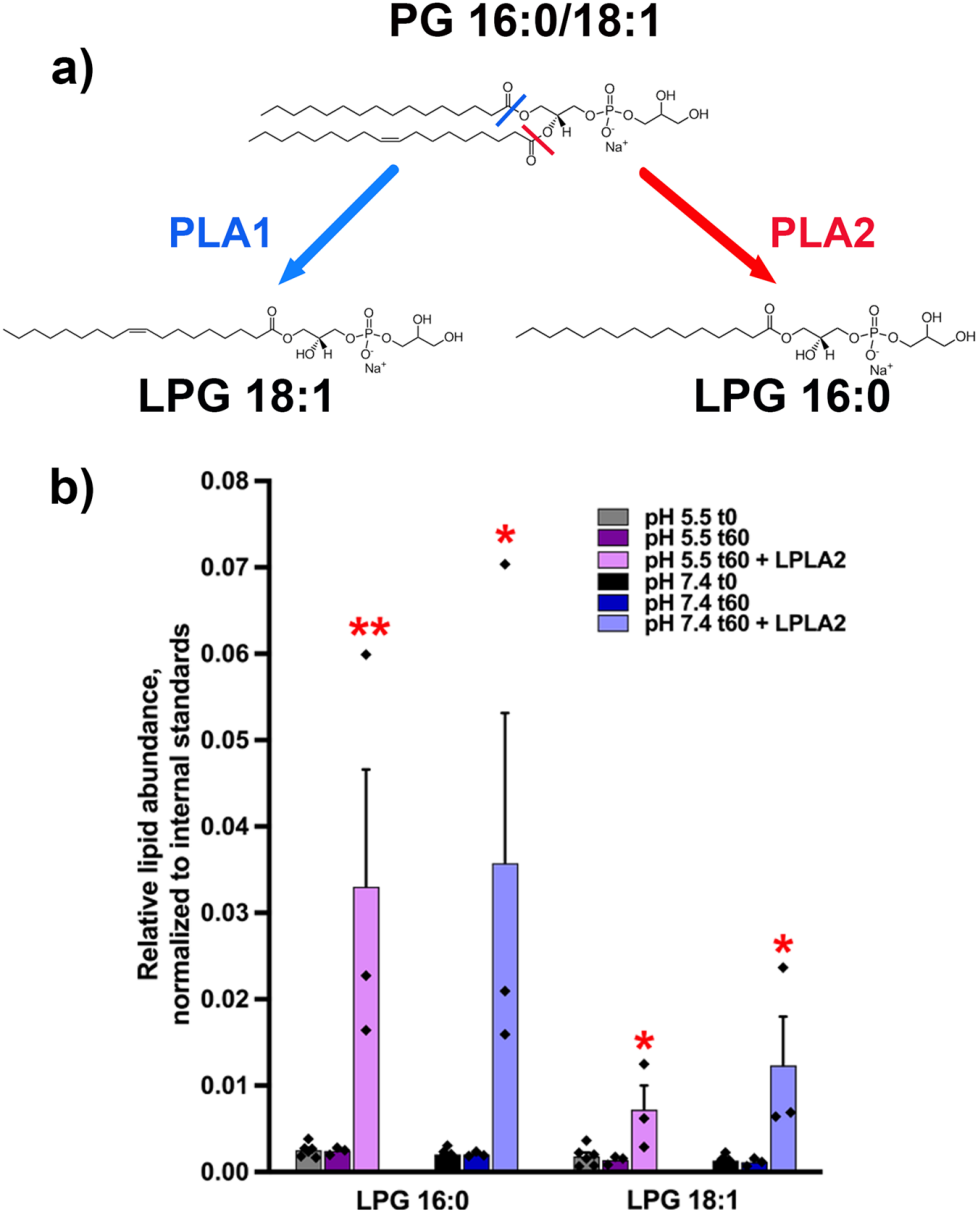
LPLA2 catalyzes the conversion of PG to LPG in vitro. **a** Experimental design. **b** LC-MS/MS results for the detection of LPG 16:0 and LPG 18:1 from PC:PG (90:10) liposomes before (t0) or after incubation at 37 °C for 1 h (t60) in the presence or absence of LPLA2 at pH 7.4 or 5.5 (*n* = 3 or 6 independent experiments for t60 and t0, respectively). * and **stand for *p* < 0.05 and *p* < 0.01, respectively, in one-way ANOVA with Dunnett’s post-test with t0 as the reference. Data are presented as mean±s.e.m.

We first tested whether the PLA2 activity of LPLA2 was sufficient to catalyze the conversion of PG to LPG, the first step in the biosynthesis of BMP. To finely dissect the activity of LPLA2, we used an in vitro system where purified recombinant LPLA2 was incubated with liposomes containing PC and PG 16:0/18:1. After incubation at 37 °C for 1 h, the lipid content of the liposomes was extracted and analyzed by liquid chromatography-mass spectrometry (LC-MS/MS) (Fig. 1). As our initial species was PG 16:0/18:1, PLA2 activity should result in detectable LPG 16:0, while PLA1 activity should result in LPG 18:1. (Fig. 1a). While no LPG was detected in the absence of LPLA2 (Fig. 1b), we observed that LPG 16:0 was generated in the presence of LPLA2 (Fig. 1b), confirming that LPLA2 displays PLA2 activity for PG, as previously suggested^31^. Some LPG 18:1 was also detected in the presence of LPLA2 (Fig. 1b), indicating that LPLA2 can have PLA1 activity towards PG. Nevertheless, in samples incubated with the enzyme, the normalized intensity of LPG 16:0 was 3–5 times higher than that of LPG 18:1 (*p* < 0.05, one sample *t*-test, theoretical mean of 1 indicating comparable intensities), suggesting that LPLA2 may have a stronger PLA2 than PLA1 activity towards PG.

We also tested the pH-sensitivity of the PLA2 activity of LPLA2. Indeed, while endoplasmic reticulum (ER) has a neutral pH (7.4), LE exhibit an acidic pH (5-5.5) that is tightly linked to their function. In order to gain insight into the functional localization of LPLA2, we tested whether the capacity of LPLA2 to convert PG into LPG is pH-dependent by incubating recombinant LPLA2 with PC:PG liposomes at a pH of 5.5 or 7.4. LPG 16:0 was detected in both pH conditions in comparable amounts (*p* = 0.907, unpaired Student’s *t* test, Fig. 1b). These results support that the enzyme has similar PLA2 activity towards PG at either pH and could thus convert PG to LPG in compartments with either a neutral or an acidic pH. Of note, the enzyme’s difference in PLA2 vs. PLA1 activity towards PG seemed exacerbated at pH 5.5 compared to pH 7.4 (*p* < 0.05, unpaired Student’s *t* test, Fig. 1b). We also controlled that no LPG was detected in the absence of PG (PC-only liposomes, Supplementary Fig. 1). Taken together, these results support that LPLA2 is sufficient to catalyse the conversion of PG to LPG in vitro.

### Modulating LPLA2 levels regulates the levels of BMP in human cell lines

We decided to further our investigation of the role of LPLA2 in BMP synthesis in a widely used mammalian cell system, i.e., human HeLa cell lines. We hypothesized that if LPLA2 is indeed regulating the limiting step in BMP synthesis, modulating the levels of LPLA2 should affect the levels of BMP.

**Fig. 2:**
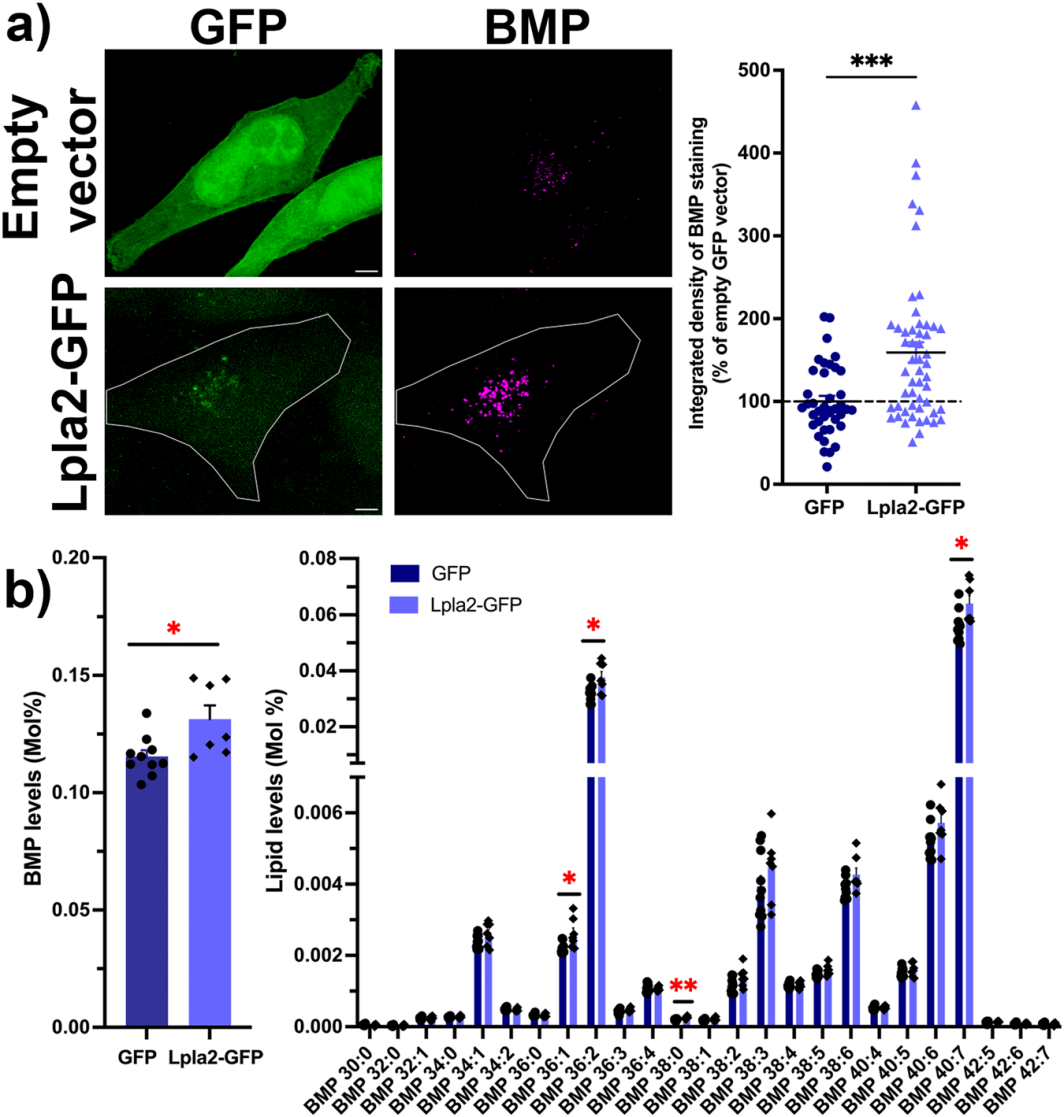
Overexpression of Lpla2 results in increased BMP levels in HeLa cells. **a** Left panel, representative maximum intensity projections of HeLa cells expressing Lpla2-GFP or GFP (green) immunostained for endogenous BMP (magenta). Scale bar, 5  μm. Right panel, quantification of integrated densities of BMP fluorescence signal in cells expressing Lpla2-GFP (159 ± 12%, *n* = 53 cells, three independent experiments) and GFP (100 ± 7%, *n* = 39 cells, three independent experiments). *** stands for *p* < 0.001 in Mann–Whitney test. **b** Quantification of overall BMP levels (left) and individual BMP species (right) by LC-MS/MS. Dark blue bars: GFP (*n* = 10 transfections); light blue bars: Lpla2-GFP (*n* = 7 transfections). * and ** stand for *p* < 0.05 and *p* < 0.01 in multiple unpaired *t*-tests with Welch’s correction, respectively. All values are given as mean ± s.e.m.

We first tested whether increasing the levels of LPLA2 would lead to increased levels of BMP. HeLa cells transiently transfected with Lpla2 fused to a green fluorescent protein (GFP) reporter (Lpla2-GFP) or with the control empty GFP vector were processed by immunocytochemistry with antibodies targeting endogenous BMP (Fig. 2a). Quantification of confocal z-stacks revealed that levels of BMP fluorescence signal were largely increased in cells overexpressing the Lpla2-GFP fusion protein compared to cells overexpressing GFP alone (159 ± 12% vs. 100 ± 7%, Fig. 2a). In parallel, the lipid profiles of HeLa cells transiently transfected with Lpla2-GFP or with the control empty GFP vector were analyzed by lipidomics. Of 34 lipid classes, 5 were affected by over-expression of Lpla2-GFP (Supplementary Fig. 2), including BMP (Fig. 2b). We observed a small but significant increase in BMP levels in cells transiently transfected with Lpla2-GFP compared to controls (114 ± 5% vs. 100 ± 2%). We also analyzed 25 individual species of BMP and found that 4 were increased in cells expressing Lpla2-GFP (BMP 36:1; BMP 36:2; BMP 38:0 and BMP 40:7; Fig. 2b). Using two independent methods, we thus demonstrated that increasing Lpla2 levels leads to increased BMP levels in mammalian cells.

**Fig. 3:**
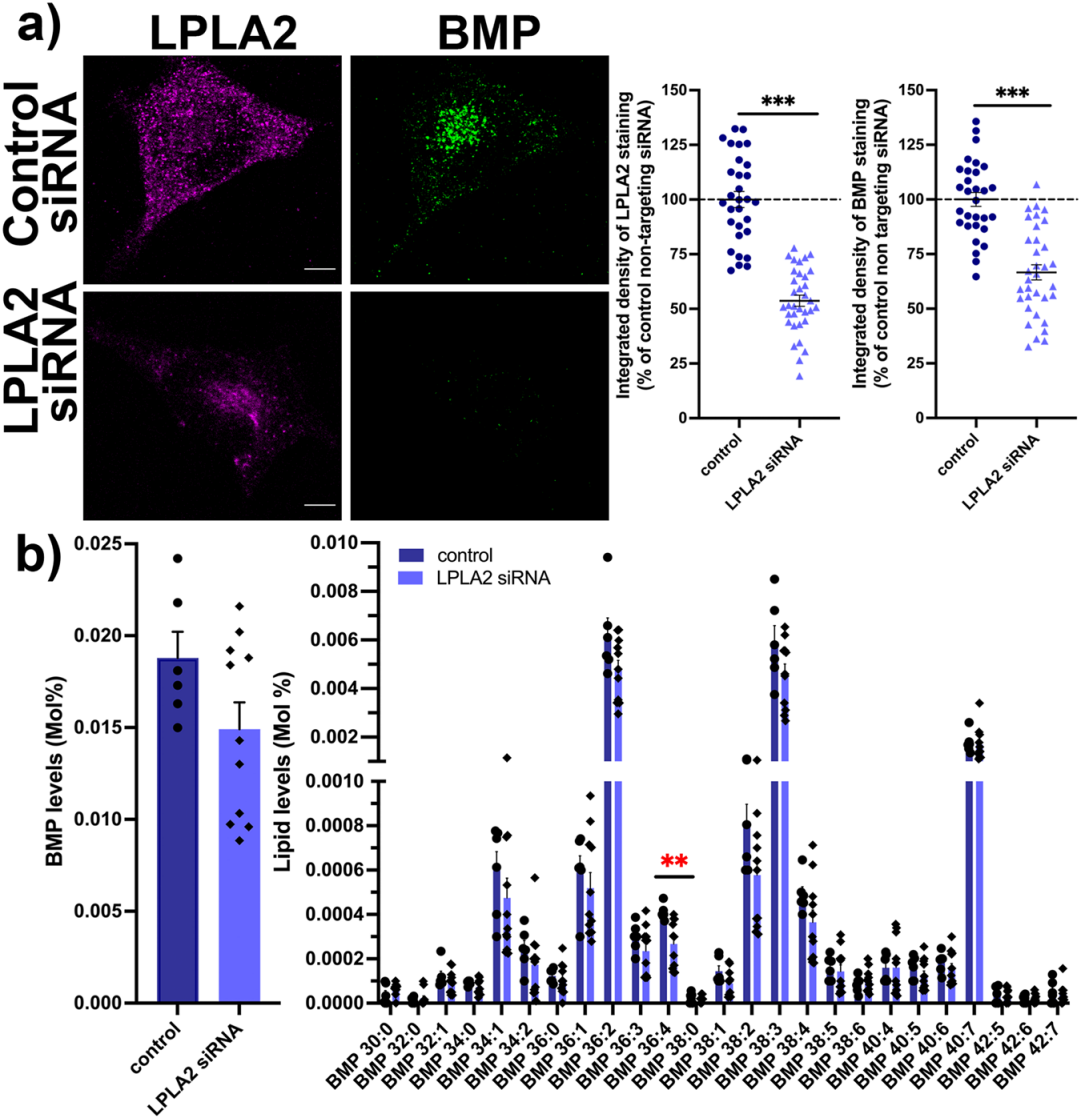
Downregulation of LPLA2 levels results in a decrease in BMP levels in HeLa cells. **a** Left panel, representative maximum intensity projections of HeLa cells treated with a control non-targeting siRNA or with an siRNA targeting LPLA2, immunostained for endogenous LPLA2 (magenta) and BMP (green). Scale bar, 5 μm. Right panel, quantification of integrated densities of LPLA2 and BMP fluorescence signals in cells treated with an siRNA targeting LPLA2 (54 ± 3% and 67 ± 3%, respectively, *n* = 34 cells, three independent experiments) or with a control non targeting siRNA (100 ± 4% and 100 ± 3%, respectively, *n* = 30 cells, three independent experiments). *** stands for *p* < 0.001 in Student’s unpaired *t*-test. **b** Quantification of overall BMP levels (left) and individual BMP species (right) by LC-MS/MS. Dark blue bars: control siRNA (*n* = 6 treatments); light blue bars: siRNA directed against LPLA2 (*n* = 11 treatments). ** stands for *p* < 0.01 in multiple unpaired *t*-tests with Welch’s correction. All values are given as mean ± s.e.m.

We further tested the reverse hypothesis, i.e., whether downregulating the levels of LPLA2 would lead to decreased BMP levels. LPLA2 protein levels were decreased using Accell siRNA knock-down (KD) in HeLa cells, a technique that does not require the use of lipid-based transfection agents. We fixed the siRNA-transfected cells and processed them for immunocytochemistry with antibodies directed towards endogenous BMP and LPLA2 (Fig. 3a). Control experiments in which the antibody was preincubated with the purified protein provided evidence that the staining observed with the LPLA2 antibody was specific (Supplementary Fig. 3). After quantification of confocal z-stacks, we observed that reducing LPLA2 levels by ~50% led to significant reduction in BMP levels (67 ± 3% *vs*. 100 ± 7% in cells treated with a non-targeting siRNA) (Fig. 3a). We also performed lipidomics analysis of HeLa cells treated with a non-targeting siRNA or with an siRNA directed against LPLA2 (Supplementary Fig. 4). BMP levels showed a trend for a decrease in LPLA2-depleted cells compared to controls (79 ± 2% vs. 100 ± 3%), but this effect did not reach statistical significance (Fig. 3b). We also analyzed 25 individual species of BMP and found that the majority of BMP species showed a trend for a decrease, with BMP 36:4 reaching statistical significance (Fig. 3b). Decreasing LPLA2 levels thus results in an overall decrease of BMP levels in mammalian cells. Altogether, our findings support that LPLA2 plays an important role in BMP biosynthesis in eukaryotic cells.

### Modulating LPLA2 levels affects BMP-regulated pathways in human cell lines

We then reasoned that if LPLA2 levels were indeed regulating BMP levels, LPLA2 levels would also impact structural and functional cellular outcomes associated with BMP levels, such as LE/Lys morphology and cholesterol levels.

**Fig. 4:**
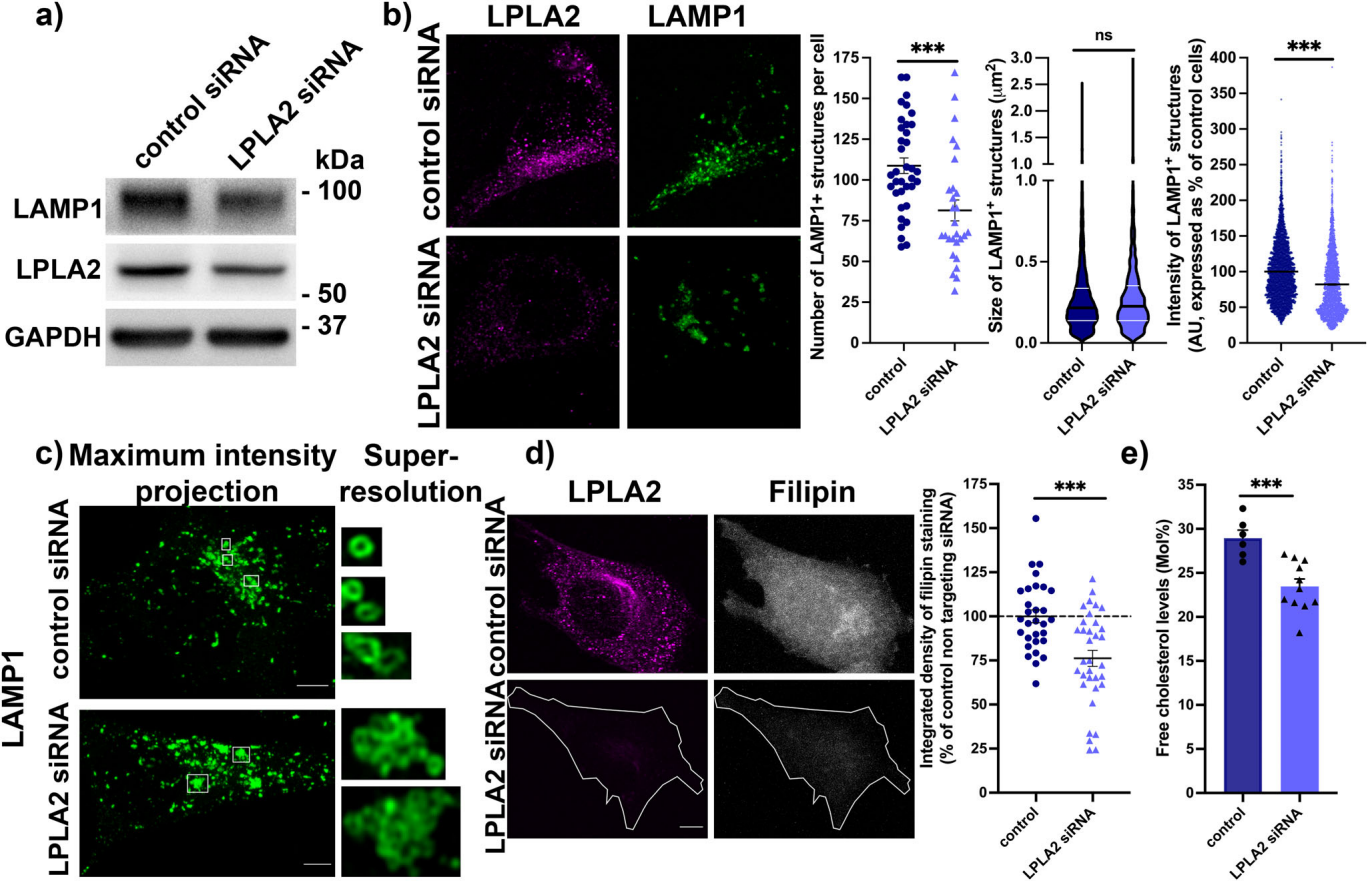
Lowering LPLA2 levels leads to aberrant LE/Lys morphology and decreased cholesterol levels in HeLa cells. **a** Western blot analysis of LAMP1 and LPLA2 levels of HeLa cells treated with a control non-targeting siRNA or with an siRNA targeting LPLA2. GAPDH was used as an equal loading marker. **b** Left panel, representative maximum intensity projections of confocal z-stacks of HeLa cells treated with a control non-targeting siRNA or with an siRNA targeting LPLA2, immunostained for endogenous LPLA2 (magenta) and LAMP1 (green). Scale bar, 5 μm. Right panel, quantification of confocal z-stacks. From left to right, scatter dot blot of the number of LAMP1-positive structures per cell in control (109 ± 5 structures, 36 cells, three independent experiments) and LPLA2 siRNA-treated cells (81 ± 6 structures, 28 cells, three independent experiments); violin plot of the size of LAMP1-positive structures in control (0.27 ± 0.003 μm^2^, 3914 puncta from 36 cells) and LPLA2 siRNA-treated cells (0.28 ± 0.005 μm^2^, 2277 puncta from 28 cells); scatter dot plot of the average fluorescence intensity of LAMP1-positive structures in control (100 ± 0.7%, 3915 puncta from 36 cells) and LPLA2 siRNA-treated cells (82 ± 0.9%, 2277 puncta from 28 cells). A.U. stands for arbitrary units, *** and ns stand for *p* < 0.001 and *p* > 0.05, respectively, in Mann–Whitney test. **c** Left, representative maximum intensity projections of confocal z-stacks of HeLa cells treated with a control non-targeting siRNA or with an siRNA targeting LPLA2, immunostained for endogenous LAMP1 (green). Scale bar, 5 μm. Right, representative single-section super-resolution (Airyscan) zoom-in. **d** Left panel, representative maximum intensity projections of HeLa cells treated with a control non-targeting siRNA or with an siRNA targeting LPLA2, immunostained for endogenous LPLA2 (magenta) and stained with filipin (gray). The cell outline is indicated by a white line in the bottom panel for clarity. Scale bar, 5 μm. Right panel, quantification of integrated densities of filipin fluorescence signals in cells treated with an siRNA targeting LPLA2 (76 ± 5%, *n* = 34 cells, three independent experiments) or with a control non targeting siRNA (100 ± 4%, *n* = 30 cells, three independent experiments). *** stands for *p* < 0.001 in Student’s unpaired *t*-test. **e** Quantification of free cholesterol levels by LC-MS/MS. Dark blue bars: control siRNA (*n* = 6 treatments); light blue bars: siRNA directed against LPLA2 (*n* = 11 treatments). *** stands for *p* < 0.001 in Student’s t-test. All values are given as mean ± s.e.m.

To test the impact of LPLA2 levels on LE/Lys, protein levels of LAMP1, a LE/Lys marker, were analyzed using western blot (Fig. 4a and Supplementary Fig. 5). We found that LAMP1 levels were significantly reduced in cells treated with siRNAs targeting LPLA2 (53 ± 11% of control cells treated with a non-targeting siRNA). In parallel, siRNA-treated cells were fixed and processed for immunocytochemistry with an antibody targeting LAMP1, to analyze LE/Lys morphology. Quantification of confocal z-stacks revealed that there were on average less LAMP1-positive structures in LPLA2-depleted cells compared to controls (Fig. 4b), and that their fluorescence was less intense (Fig. 4b). Overall, the size of LAMP1-positive structures was comparable in LPLA2-depleted cells compared to controls (Fig. 4b). However, when using super-resolution microscopy (AiryScan), we observed that LAMP1-positive structures presented with an altered morphology in LPLA2-depleted cells compared to controls (Fig. 4c). Indeed, while LAMP1-positive structures have a small round individual vesicular morphology in control cells, they seemed to present with an aberrant intra-lumenal accumulation of lysosomal membrane in LPLA2 KD cells.

We further tested the impact of LPLA2 levels on cholesterol levels. It has been reported that decreasing BMP levels, using an siRNA directed against Alix, leads to decreased cellular cholesterol levels^7^. We fixed siRNA-treated cells and processed them for staining with filipin, a fluorescent antibiotic that recognizes free cholesterol. We found that in cells showing reduced levels of LPLA2, where BMP levels were also reduced (Fig. 3a), cholesterol levels were significantly decreased (76 ± 5% compared to 100 ± 4% in control siRNA-treated cells scramble) (Fig. 4d). These latter findings were also confirmed by LC-MS/MS, as we observed a ~20% reduction in free cholesterol levels in siRNA-treated cells compared to controls (Fig. 4e and Supplementary Fig. 4). Overall, these findings support that modulating LPLA2 levels impact BMP-related pathways such as LE/Lys homeostasis and cholesterol storage.

### Increasing LPLA2 levels alleviates cholesterol accumulation in NPC fibroblasts

**Fig. 5:**
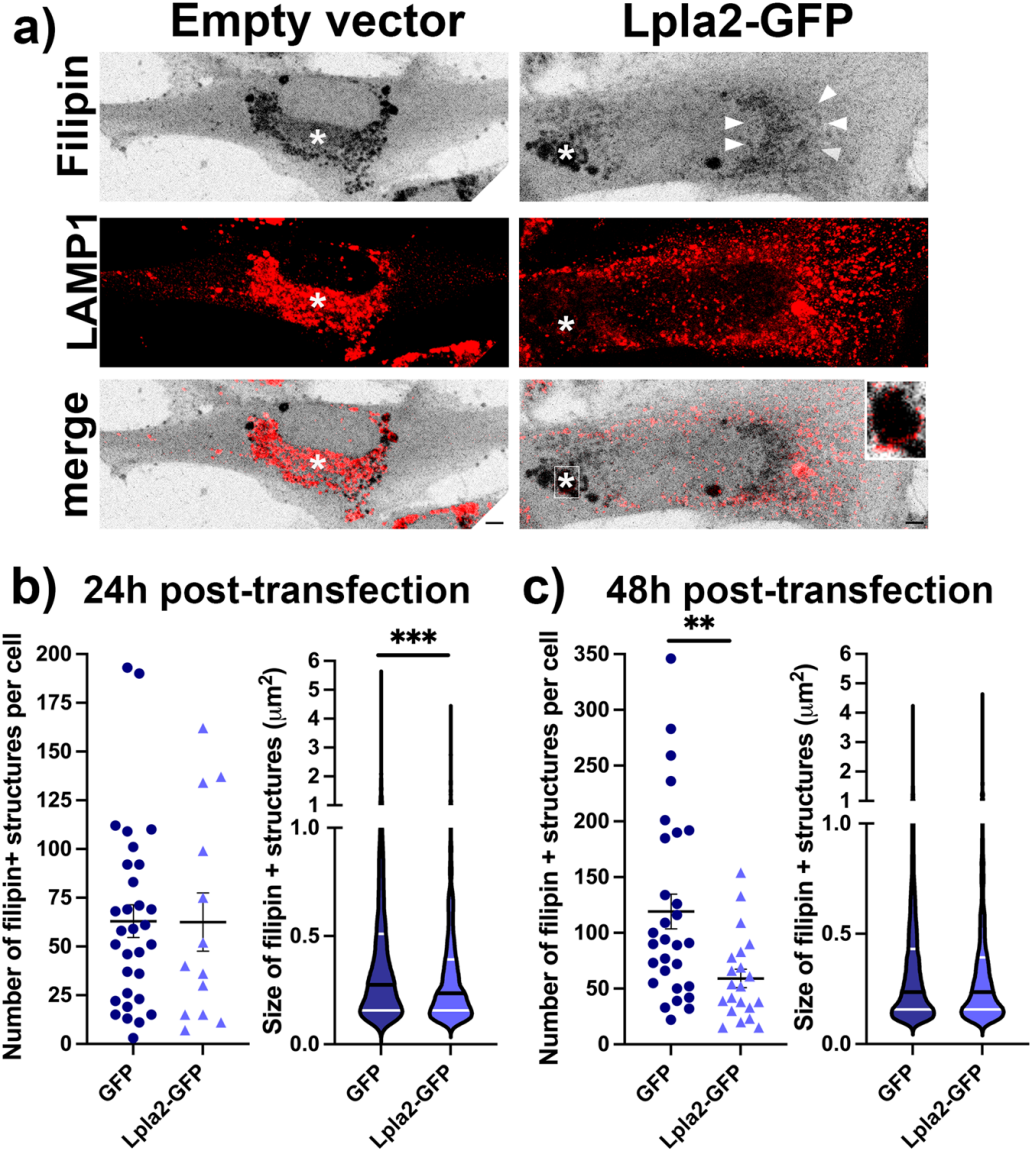
Over-expressing Lpla2 in human NPC fibroblasts leads to improved cholesterol accumulation phenotype. **a** Representative maximum intensity projections of human NPC fibroblasts expressing GFP or Lpla2-GFP stained with filipin (gray) and immunolabeled for LAMP1 (red). Asterisks indicate filipin puncta, while arrowheads indicate a more diffuse perinuclear filipin staining. Scale bar, 5 μm. **b** Quantification of the number and size of filipin-positive structures in cells expressing GFP or Lpla2-GFP for 24 h. Left, scatter dot blot of the number of filipin-positive structures per cell in GFP- (63 ± 8 structures, 31 cells, 3 independent experiments) and Lpla2-GFP-expressing cells (63 ± 15 structures, 13 cells, 3 independent experiments); violin plot of the size of filipin-positive structures in GFP- (0.45 ± 0.01 μm^2^, 1952 puncta from 31 cells) and Lpla2-GFP-expressing cells (0.35 ± 0.01 μm^2^, 813 puncta from 13 cells). *** stands for *p* < 0.001 in Mann–Whitney test. **c** Quantification of the number and size of filipin-positive structures in cells expressing GFP or Lpla2-GFP for 48 h. Left, scatter dot blot of the number of filipin-positive structures per cell in GFP- (119 ± 16 structures, 29 cells, 3 independent experiments) and Lpla2-GFP-expressing cells (59 ± 8 structures, 21 cells, 3 independent experiments); violin plot of the size of filipin-positive structures in GFP- (0.36 ± 0.01 μm^2^, 3454 puncta from 29 cells) and Lpla2-GFP-expressing cells (0.37 ± 0.01 μm^2^, 1241 puncta from 21 cells). ** stands for *p* < 0.01 in unpaired *t*-test with Welch’s correction. All values are given as mean ± s.e.m.

Mutations in the *NPC1* or *NPC2* genes cause Niemann–Pick Type C (NPC) disease, a fatal neurodegenerative lysosomal storage disorder associated with accumulation of cholesterol and other lipids in the endolysosomal system^32^. While levels of BMP and cholesterol shift in parallel in non-diseased cells (e.g., Fig. 4d, e), their relationship is different in models of disease. In particular, it has been proposed that BMP becomes limiting in cellular models of NPC, and that increasing BMP levels would improve the cholesterol accumulation phenotype in these models^7^. Indeed, supplementing human NPC fibroblasts with exogenous BMP^7^, exogenous PG^8^ or thioperamide maleate, an inverse agonist of the histamine H3 receptor HRH3 that selectively increases BMP levels^33^, leads to reduced cholesterol accumulation. We thus hypothesized that over-expressing Lpla2-GFP in human NPC fibroblasts may have a beneficial effect on cholesterol accumulation in LE/Lys. Human NPC1-deficient fibroblasts transiently transfected with Lpla2-GFP or with the control empty GFP vector were stained with filipin (Fig. 5a). The NPC fibroblasts expressing GFP showed a typical pattern of filipin puncta overlapping with LAMP1 staining, in good accordance with the expected accumulation of cholesterol in the endolysosomal system. Interestingly, NPC fibroblasts expressing Lpla2-GFP showed a mixed filipin distribution. Indeed, filipin labeled not only dense puncta (indicated here by an asterisk) rimmed with LAMP1-positive membranes (Fig. 5a, inset) but also a more diffuse perinuclear region (indicated by arrowheads) that was not LAMP1-positive. Quantification of confocal z-stacks after 24 h of transfection revealed that while the number of filipin-positive puncta was unchanged, their size was significantly decreased in cells expressing Lpla2-GFP (Fig. 5b), suggesting the beginning of an improvement of the lysosomal cholesterol accumulation in these cells. After 48 h of transfection, quantification of confocal z-stacks indicated that the number of filipin-positive puncta per cell was reduced by half in cells expressing Lpla2-GFP (Fig. 5c), supporting that the lysosomal cholesterol accumulation phenotype was alleviated in these cells. The remaining filipin puncta had a size similar to the control cells (Fig. 5c). Altogether, these results indicate that increasing LPLA2 levels has a beneficial effect on the LE/Lys cholesterol accumulation phenotype in NPC cells.

## Discussion

In this study, a significant advancement was made by identifying LPLA2 as one of the biosynthetic enzymes involved in the de novo synthesis of BMP from PG. Here we confirm that LPLA2 is sufficient to catalyze the conversion of PG to LPG in vitro. We also demonstrate that LPLA2 levels bidirectionally correlate with BMP levels in human cell lines, strongly suggesting that LPLA2 plays a key functional role in the biosynthesis of this lipid. We show that these changes in BMP levels are accompanied by changes in well-described downstream pathways, such as LE/Lys homeostasis and cholesterol levels. Finally, we establish the disease-relevance of our findings by showing that expressing LPLA2 in human NPC fibroblasts can alleviate their cholesterol accumulation phenotype. Collectively, our results strongly support that LPLA2 plays an important role in BMP synthesis, by regulating its limiting first step.

Using liposomes and LC-MS/MS, we demonstrated that purified active LPLA2 displays PLA2 activity towards PG in vitro. Interestingly, the resulting product, LPG, was detected in comparable amounts after a one-hour incubation of LPLA2 and PG either at neutral or at acidic pH. The conversion of PG to LPG by LPLA2 thus seems to not be strongly pH-dependent within this timeframe; however, we cannot rule out that there may be pH-related differences in the kinetics of the PG to LPG conversion that were not detectable in our assay.

We also showed that overexpression of Lpla2 leads to increased levels of BMP, as demonstrated both by immunofluorescence and by LC-MS/MS. In addition to increased BMP levels, lipidomic analysis revealed that overexpression of Lpla2-GFP also resulted in an increase of diacylglycerol (DG) and phosphatidic acid (PA) and a decrease in plasmogen lysophosphatidylethanolamine (LPEp) and N-Acyl Serine (NSer), compared to overexpression of GFP only. Although small in effect size, the fact that both DG and PA were increased is interesting, as these two lipids are metabolically linked. Indeed, DG can be converted to PA by the action of diacylglycerol kinases, while PA can be converted to DG through lipin activity^34^. Lpla2 might stimulate the activity of other lipid enzymes, such as phospholipases C (conversion of phosphatidylcholine or phosphatidylinositol 4,5-bisphosphate to DG) or phospholipases D (conversion of phosphatidylcholine to PA). Of note, diacylglycerol kinase α activity has been linked to the formation of mature MVBs and the secretion of exosomes in T lymphocytes^35^, suggesting a possible functional connection between levels of BMP, DG, and PA.

Conversely, we observed that decreasing LPLA2 levels resulted in decreased levels of BMP, as demonstrated both by immunofluorescence and by LC-MS/MS. As expected, LC-MS/MS results displayed more variability than imaging results did. This is likely due to the fact that while imaging focuses on one population of cells (with decreased LPLA2), LC-MS/MS results correspond to the average of multiple populations of cells (with varying levels of LPLA2). In addition to decreased BMP 36:4 levels, lipidomic analysis of LPLA2-depleted cells revealed multiple changes compared to controls, including decreased levels of free cholesterol (FC) and phosphatidylethanolamine (PE) and increased levels of alkyl phosphatidylcholine (PCe), sphingomyelin (SM), Acyl Carnitine (AC) and dihydrosphingomyelin (dhSM). This decrease in free cholesterol was confirmed by filipin staining and is in good accordance with the proposed role of BMP in the control of cellular cholesterol levels^7^. Similarly, the observed increase in levels of SM and dhSM corroborates the proposed involvement of BMP in the degradation of sphingolipids^4^.

Levels of BMP also profoundly impact the morphology of LE/Lys. Indeed, we observed modifications in the number and morphology of LAMP1-positive LE/Lys. The aberrant LAMP1 membranes observed after decreasing LPLA2 and BMP levels are reminiscent of the inner membrane disorganization that was reported after internalization of an antibody directed against BMP^1^. This suggests that decreasing BMP levels or sterically hindering its accessibility with an antibody interferes with its function in the proper formation of ILVs, either by disrupting its interaction with Alix^3^, by inhibiting the formation of BMP clusters proposed to play an essential role in the release of ILVs from the limiting membrane^36^ or both. However, we cannot fully rule out the alternative possibility that decreasing BMP levels leads to the formation of clusters of aggregated individual LE/Lys.

Altogether, our results strongly support that LPLA2 plays a central role in the biosynthesis of BMP, and consequently on BMP-dependent pathways. An important issue that remains unsolved is the source of the PG used to synthesize BMP. Indeed, PG is mostly found in mitochondria^37^, but not in LE/Lys. As proposed by others^23,38,39^, the two most likely sources of PG are autophagy/mitophagy and inter-organelles contact sites between LE/Lys and a PG-containing organelle. This will require further investigation.

Interestingly, it has been reported that anionic phospholipids, and in particular BMP, can stimulate PLA2 enzymatic activity^40^, suggesting that there could be a positive feedback loop at play that enables both the continual synthesis of BMP and the increased catabolism of other lipids by LPLA2 and other lipases in the LE/Lys. Indeed, the unique sn-1:sn-1’ configuration of BMP makes it a poor substrate for phospholipases^39^ while its negative charge at acidic pH makes it a docking site for lipid binding and lipid degrading enzymes, thus facilitating lipid catabolism^4^.

Of note, *Pla2g15*^*−/−*^ mice have been reported in the literature^41^. In these mice, exon 5 of *Pla2g15*—which codes for the lipase motif—was deleted, leading to an absence of PLA2 or 1-O-acylceramide synthase activity. The lifespan and fertility of *Pla2g15*^*−/−*^ mice were unaffected, yet they showed splenomegaly, as well as an increased number of enlarged lung alveolar macrophages that accumulated phospholipids^41^. This model was also used to propose a role of LPLA2 in host immunity to respiratory pathogenic bacteria^42^. Another *Pla2g15*^*−/−*^ mouse model supported that LPLA2 could also play a role in atherosclerosis^43^. To our knowledge, BMP levels have not been investigated in *Pla2g15*^*−/−*^ mice. However, it is interesting to note that levels of its precursor PG were found to be increased in one of these models^41^, in good accordance with our hypothesis.

While LPLA2 is ubiquitously expressed, it is highly enriched in alveolar macrophages and has a 50-fold higher activity in these cells than in any other cell type^31,44^. Interestingly, while BMP typically accounts for only 1–2% of total phospholipids, it is highly enriched in alveolar macrophages, representing up to 17% of all phospholipids^45^, drawing parallels between LPLA2 activity and BMP levels and supporting our hypothesis that LPLA2 plays a key role in BMP synthesis.

Very recently, LPLA2 was a hit in two genome-wide CRISPR screens designed to identify genes that, when knocked out, alter levels of cholesterol or BMP^46^. Although the function of LPLA2 was not explored further in this report, these results are in good accordance with our findings.

Moreover, the expression of LPLA2 has been proposed to be regulated by an RXR-dependent pathway^47^. It is also well established that the LXR/RXR pathway acts as a cholesterol sensor and regulates intracellular cholesterol metabolism^48^. This suggests that there could be a transcriptional component to the cholesterol/BMP relationship, through control of LPLA2 expression.

We also provide evidence that expressing Lpla2 in a cellular model of NPC disease is sufficient to alleviate the LE/Lys cholesterol accumulation phenotype that is typical of this disorder. We observe that the filipin-positive puncta first decrease in size and then in number, supporting the known beneficial effect of BMP in NPC models^7,8,33^, and highlighting the potential of LPLA2 as a therapeutic target for NPC. As BMP and BMP-dependent pathways are involved in a vast array of disorders, we anticipate that modulation of LPLA2 levels, activity, or both, could be of therapeutic relevance in a number of these diseases.

Our findings, taken together, provide strong evidence that LPLA2 is one of the elusive enzymes regulating the first step of BMP biosynthesis first alluded to several decades ago. We thus believe that we are strongly contributing to a long-standing question in the field of cell biology with implications for intracellular trafficking, lysosomal degradation, intercellular communication, and associated disorders stemming from the dysfunction of these pathways. Mouse models in which LPLA2 is selectively targeted^41,43^ as well as LPLA2-selective inhibitors^31^ already exist, yet it is likely that additional models and compounds will be developed in the near future. Based on our findings, we expect that present and future models and compounds will provide genetic and pharmacological tools to assess the contribution of LPLA2 and BMP to pathogenic mechanisms of viral infection and neurodegeneration, and pave the way for potential therapeutic avenues for these disorders.

## Methods

### Reagents

Lipids were obtained from Avanti Polar Lipids. HEPES, KCl, MES, dimethyl sulfoxide (DMSO), filipin, sucrose, bovine serum albumin (BSA) were from Sigma-Aldrich. Saponin was from Acros. Dulbecco’s Modified Eagle Medium (DMEM), Minimum Essential Media (MEM), Hank’s balanced salt solution (HBSS), and Phosphate buffered saline (PBS) were from Gibco.

### Antibodies and recombinant proteins

Recombinant active human lysosomal phospholipase A2 was obtained from Echelon Biosciences. The antibodies were obtained from the following sources: rabbit antibodies to LAMP1 (Abcam, 1/300 in immunofluorescence (IF), 1/500 for western blots (WB)), LPLA2 (Novus Biologicals, 1/500 for WB); a rat monoclonal antibody (mAb) to LPLA2 (Echelon Biosciences, 1/400 for IF) and mouse mAbs to BMP (Echelon Biosciences, 1/100 for IF) and GAPDH (EnCor Biotechnology, 1/2000 for WB). Peroxidase-conjugated secondary antibodies were from Biorad and Alexa-conjugated fluorescent secondary antibodies were from Life Technologies.

### Liposome preparation and lipid extraction

Lipids used were PC 16:0/18:0 and PG 16:0/18:1 PG at 10 mg/mL in chloroform (Avanti Polar Lipids). All experiments were performed protected from light. On day one, PC was either used alone or mixed with PG in a 90/10 ratio for a final volume of 50 μL. The resulting lipid mixes were vortexed for 10 s and centrifuged for 1 min at 16,100 × *g*. Lipids were then dried using a SpeedVac vacuum concentrator. The resulting dried lipids were resuspended in 100 μL of buffer A (HEPES 30 mM, KCl 200 mM, pH 7.4) or buffer B (MES 30 mM, KCl 200 mM, pH 5.5) and sonicated in a water bath sonicator for 10 min to help with the formation of multilamellar vesicles/liposomes (crude liposomes). The resulting solutions were stored overnight at 4 °C. The next day, liposomes were sonicated for 10 min and placed on ice. They were then diluted 1/100 in buffer A or B to prepare the working solution. For t0 conditions, lipids were extracted right away. For t60 conditions, lipids were extracted after incubation for 1 h at 37 °C with shaking. For t60 + LPLA2 conditions, lipids were extracted after incubation for 1 h at 37 °C with shaking in the presence of recombinant active human lysosomal phospholipase A2 (Echelon Biosciences, with a LPLA2: PG molar ratio of 1/10). All buffers and tubes used for lipid extraction were pre-chilled at 4 °C. Lipids were extracted from each initial 50 μL reaction volume using 900 μL chloroform:methanol (5:4). The resulting solution was vortexed for 10 sec and supplemented with 200 μL KCl 1 M. The resulting solution was vortexed for 30 s and incubated on ice for 1 min. This step was repeated twice. The solution was then centrifuged for 2 min at 16,100 × *g*. The organic layer was then transferred to a new tube, dried using a SpeedVac vacuum concentrator and stored at −80 °C.

### Analysis of phospholipids and lysophospholipids in liposomes

Dried lipid extracts were re-suspended in 50 μL of chloroform/methanol (C/M) 2/1 v/v. 10 µL of these solutions were transferred to mass spectrometry vials and diluted 1/1 v/v with C/M 2/1 containing 10 µg/mL PC 14:0/14:0, 10 µg/mL LPC 17:0, 2 µg/mL PG 14:0/14:0, 2 µg/mL LPG 14:0, 2 µg/mL PS 14:0/14:0, and 2 µg/mL LPS 17:0. 2 µL of each sample were pooled together to make a quality control (QC) sample for instrument response stability monitoring. These solutions were used directly for LC-MS/MS analysis, without further dilution. In addition, the QC samples were also diluted 2×, 4×, 8×, and 16× with C/M 2/1 v/v for response linearity monitoring. Chromatographic separation was achieved on an Agilent 1290 Infinity Binary Pump, using hydrophilic interaction liquid chromatography (HILIC) on a Kinetex HILIC column (2.1 mm internal diameter, 100 mm length, 2.6 µm particle size, 100 Å pore size). Gradient elution used solvents A (95% Acetonitrile/5% 25 mM ammonium acetate pH 4.6) and B (50% Acetonitrile/50% 25 mM ammonium acetate pH 4.6) as follows: from 1% to 25% B in 6 min, from 25% to 90% B in 1 min, back to 1% B in 0.1 min, keep at 1% B for 3 min (runtime: 10.1 min). The flow rate was 0.5 mL/min and the column temperature 30 °C. Quantification of PC, LPC, PG, and LPG was accomplished on an Agilent 6490 triple quadrupole mass spectrometer using multiple reaction monitoring (MRM) transitions under both positive (headgroup-specific transitions) and negative (fatty acyl-specific transitions) electrospray ionization (ESI) with the following parameters: gas temperature, 200 °C; gas flow, 12 L/min; capillary voltage, 3500 V. 10 QC samples were first analyzed to stabilise instrument response. When instrument stability was satisfactory, the analytical sequence was started. Blank and QC were injected every 11 sample injections to monitor the stability of the instrument response. QC dilution series was used to check linearity of response. To be quantified in the samples, an MRM transition must pass the following criteria: average signal in blank lower than 10% average signal in pooled QC, RSD < 25% over all the QC, Pearson *r*^2^ > 0.9 in QC dilution series. Quantification data was extracted using Agilent MassHunter Quantitative Analysis (QQQ) software version B.08. The data were manually curated to ensure that the software integrated the right peaks. Areas under curve (AUC) of the extracted ion chromatograms peaks for each MRM transition were extracted to Microsoft Excel.

### Cell cultures

HeLa cells were maintained at 37 °C in a humidified 5% CO_2_ atmosphere in DMEM with Glutamax supplemented with 10% fetal bovine serum and 1% penicillin/streptomycin (all from Life technologies). Human NPC1-deficient fibroblasts (GM18453) were obtained from the Coriell Institute and maintained at 37 °C in a humidified 5% CO_2_ atmosphere in MEM supplemented with 15% fetal bovine serum and 1% penicillin/streptomycin (all from Life technologies). Cells were negative for mycoplasma contamination.

### Plasmids and RNA interference

Lpla2-GFP and the corresponding empty GFP vector were obtained from Origene (MG225901 and PS100010). HeLa cells and NPC fibroblasts were transiently transfected using Lipofectamine LTX (Life Technologies) and protein expression was assessed after 24 h (HeLa cells, NPC fibroblasts) or after 48 h (NPC fibroblasts). For KD experiments, Accell Human PLA2G15 siRNA SMARTpool and Accell Non-targeting Pool were obtained from Horizon Discovery. HeLa cells were treated with Accell Human siRNA using Accell siRNA delivery media (Horizon) as per the manufacturer’s instructions and LPLA2 knock-down was assessed at 120 h.

### Lipidomics

Lipidomics profiling was performed using Ultra Performance Liquid Chromatography-Tandem Mass Spectrometry (UPLC-MS/MS)^49,50^. Briefly, lipid extracts were prepared from cell lysates spiked with appropriate internal standards using a modified Bligh and Dyer method, and analyzed on a platform comprising Agilent 1260 Infinity HPLC integrated to Agilent 6490A QQQ mass spectrometer controlled by Masshunter v 7.0 (Agilent Technologies). Glycerophospholipids and sphingolipids were separated with normal-phase HPLC as described before^18^, with a few modifications. An Agilent Zorbax Rx-Sil column (2.1 mm internal diameter, 100 mm length, 1.8 µm particle size) maintained at 25 °C was used under the following conditions: mobile phase A (chloroform: methanol: ammonium hydroxide, 89.9:10:0.1, v/v) and mobile phase B (chloroform: methanol: water: ammonium hydroxide, 55:39:5.9:0.1, v/v); 95% A for 2 min, decreased linearly to 30% A over 18 min and further decreased to 25% A over 3 min, before returning to 95% over 2 min and held for 6 min. Separation of sterols and glycerolipids was carried out on a reverse phase Agilent Zorbax Eclipse XDB-C18 column (4.6 mm internal diameter, 100 mm length, 3.5 µm particle size) using an isocratic mobile phase, chloroform, methanol, 0.1 M ammonium acetate (25:25:1) at a flow rate of 300 μL/min. Quantification of lipid species was accomplished using multiple reaction monitoring (MRM) transitions^18,51,52^ under both positive and negative ionization modes in conjunction with referencing of appropriate internal standards: PA 14:0/14:0, PC 14:0/14:0, PE 14:0/14:0, PG 15:0/15:0, PI 17:0/20:4, PS 14:0/14:0, BMP 14:0/14:0, APG 14:0/14:0, LPC 17:0, LPE 14:0, LPI 13:0, Cer d18:1/17:0, SM d18:1/12:0, dhSM d18:0/12:0, GalCer d18:1/12:0, GluCer d18:1/12:0, LacCer d18:1/12:0, D7-cholesterol, CE 17:0, MG 17:0, 4ME 16:0 diether DG, D5-TG 16:0/18:0/16:0 (Avanti Polar Lipids). Lipid levels for each sample were calculated by summing the total number of moles of all lipid species measured by all three LC-MS methodologies, and then normalizing the total to mol %. The final data are presented as mean mol %.

Lipid abbreviations are as follows: (FC) Free Cholesterol, (CE) Cholesterol Ester, (AC) Acyl Carnitine, (MG) Monoacylglycerol, (DG) Diacylglycerol, (TG) Triacylglycerol, (Cer) Ceramide, (dhCer) Dihydroceramide, (SM) Sphingomyelin, (dhSM) Dihydrosphingomyelin, (Sulf) Sulfatide, (MHCer) Monohexosylceramide (galactosylceramide + glucosylceramide), (LacCer) Lactosylceramide, (GM3) Monosialodihexosylganglioside, (GB3) Globotriaosylceramide, (PA) Phosphatidic acid, (PC) Phosphatidylcholine, (PCe) Alkyl phosphatidylcholine, (PE) Phosphatidylethanolamine, (PEp) Plasmalogen phosphatidylethanolamine, (PS) Phosphatidylserine, (PI) Phosphatidylinositol, (PG) Phosphatidylglycerol, (BMP) Bis(monoacylglycero)phosphate, (AcylPG) Acyl Phosphatidylglycerol, (LPC) Lysophosphatidylcholine, (LPCe) Alkyl lysophosphatidylcholine, (LPE) Lysophosphatidylethanolamine, (LPEp) Plasmogen lysophosphatidylethanolamine, (LPI) Lysophosphatidylinositol, (LPS) Lysophosphatidylserine, (NAPE) N-Acyl Phosphatidylethanolamine, (NAPS) N-Acyl Phosphatidylserine, (NSer) N-Acyl Serine.

### Immunocytochemistry and filipin staining

Immunofluorescence experiments were performed as in ref. ^53^. Briefly, cells grown on glass coverslips were washed once with HBSS and fixed with a solution of 4% paraformaldehyde (Electron Microscopy Sciences) and 4% sucrose (Sigma) in HBSS for 20 min at room temperature. Cells were then washed twice in HBSS and incubated with NH4Cl (50 mM in HBSS) for 10 min. Cells were then washed twice in PBS and permeabilized with solution A (saponin 0.1%, BSA 1% in PBS) for at least 45 min at 37 °C. They were then incubated with primary antibodies diluted in solution A overnight at 4 °C. The next day, cells were washed three times in solution A and incubated with fluorescent secondary antibodies diluted in solution A for 1 h at room temperature. When appropriate, filipin (0.5 mg/mL) was added to the secondary antibodies solution. Cells were then washed again three times in solution A. Cells were finally washed once with PBS and coverslips were mounted in Vectashield mounting medium (Vectorlabs).

### Confocal and super-resolution microscopy

Z-stacks were acquired by confocal laser scanning microscopy (Zeiss LSM 800). Fluorescence was collected with a 63× plan apochromat immersion oil objective (NA 1.4). When appropriate, the AiryScan module of the confocal microscope was used to generate higher-resolution z-stacks. Extraction of single z-frame and maximum intensity projections were performed with ImageJ software. Integrated densities, i.e., cell area × [(mean cell intensities summed on the whole z-stack)-(mean background intensities summed on the whole z-stack)], were measured with Image J software. The number, size, and intensity of puncta were quantified with ICY software^54^, as in^53,55^.

### Protein biochemistry and immunoblotting

Cells were washed with ice-cold HBSS, scraped, and centrifuged twice for 10 min at 16,000 × *g* at 4 °C. Pellets were resuspended in RIPA buffer (Pierce) complemented with cOmplete protease inhibitor cocktail (Roche) and proteins were extracted on a wheel at 4 °C for at least 30 min. Samples were then centrifuged for 15 min at 16,000 × *g* and proteins in the supernatant were processed for protein dosage (BCA, Pierce). SDS–PAGE was performed on samples (10-15μg total proteins) loaded in NuPAGE 4–12% Bis-Tris gels (Life technologies). Wet transfer was performed at 80 V for 1 h 45 min at 4 °C (Bio-Rad). Primary and secondary antibodies were incubated overnight at 4 °C and 1h at room temperature, respectively. Revelation was performed with Immobilon Western Chemiluminescent HRP Substrate (EMD Millipore) and the chemiluminescent signal was imaged with ImageQuant LAS4000 mini (GE Healthcare). Quantification was performed with ImageJ. Uncropped blots are shown in Supplementary Fig. 4.

### Statistics and reproducibility

Statistical calculations were performed using GraphPad Prism software (version 9.2.0). All the data are given as mean ± s.e.m. In most cases, when comparing two samples, two-tailed Student’s *t*-test was performed. If a clear hypothesis was postulated that made it valid, then we used a one-tailed *t*-test. When variances were not comparable, Welch’s correction was applied. When the distribution could not be assumed to be Gaussian (e.g., for compartments size), we used a non parametric Mann–Whitney test. When more samples were compared and Bartlett’s test showed that variances could be compared, we used one-way ANOVA with Dunnett’s post-test. If variances could not be compared (*P* value in Bartlett’s test < 0.05), then we used multiple *t*-tests. Outliers, defined as values that were superior to (mean+3 standard deviations) or inferior to (mean−3 standard deviations) were excluded.

### Reporting summary

Further information on research design is available in the Nature Portfolio Reporting Summary linked to this article.

## Supplementary information

Supplemental Figures

Reporting Summary

## Data Availability

All data generated or analyzed during this study are included in this published article (and its supplementary information files).
