# Supplementary material for: Lysosomal phospholipase A2 contributes to the biosynthesis of the atypical late endosome lipid bis(monoacylglycero)phosphate
Source: Commun Biol. 2023 Feb 23;6:210. doi: 10.1038/s42003-023-04573-z (PMC9950130; doi:10.1038/s42003-023-04573-z)
Supplement: Supplementary file 1 — Supplemental Figures [file 42003_2023_4573_MOESM1_ESM.pdf]

Supplementary Figures

Lysosomal phospholipase A2 contributes to the biosynthesis of the atypical late endosome lipid bis(monoacylglycero)phosphate

Jacinda Chen, Amaury Cazenave-Gassiot, Yimeng Xu, Paola Piroli, Robert Hwang Jr., Laura DeFreitas, Robin Barry Chan, Gilbert Di Paolo, Renu Nandakumar, Markus R. Wenk, Catherine Marquer

Supplementary Figure 1

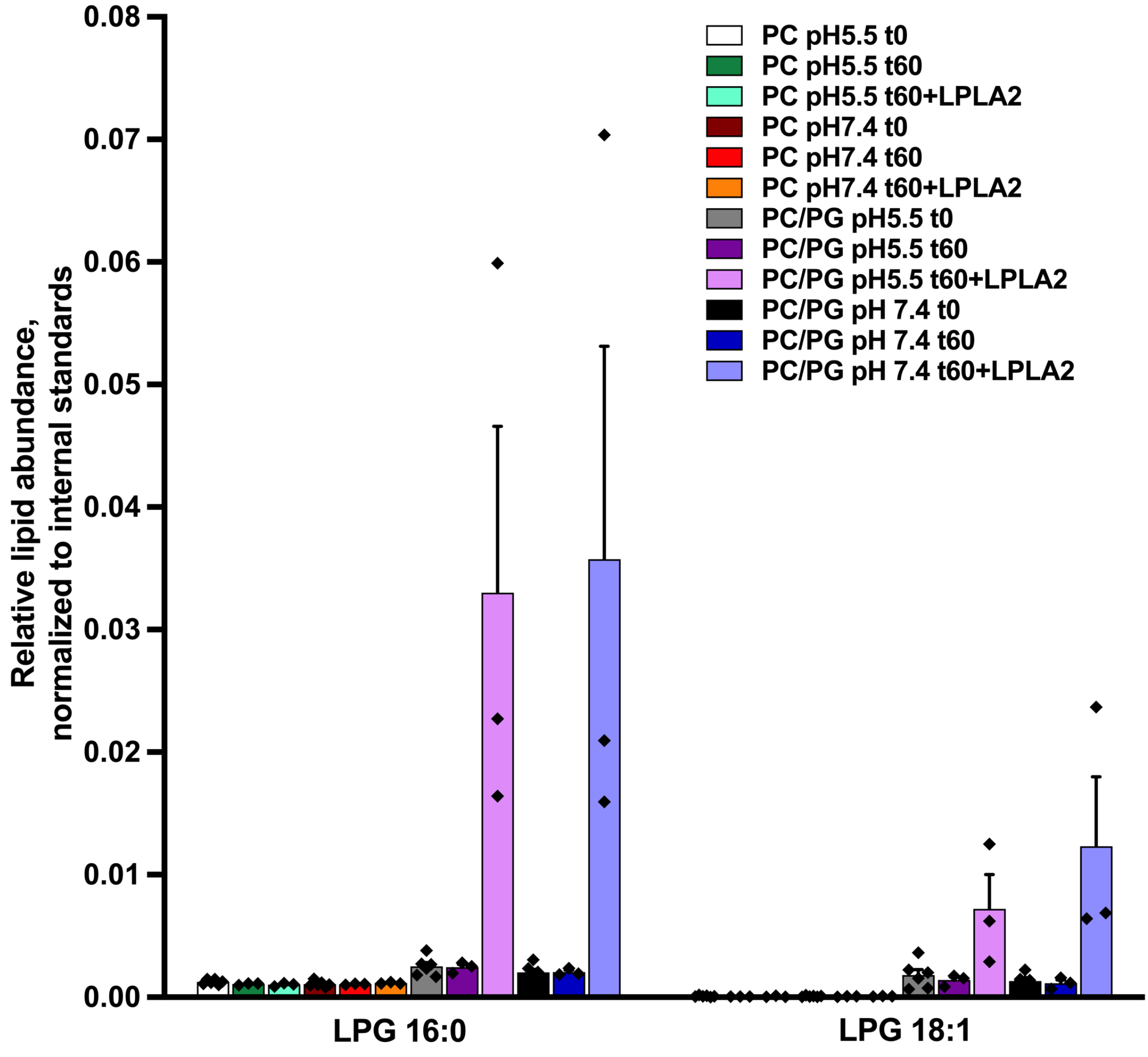

Supplementary Figure 1. LPLA2 requires PG to produce LPG *in vitro*. LC/MSMS results for the detection of LPG 16:0 and LPG 18:1 from PC liposomes before (t0) or after incubation at 37°C (t60min) in the presence or absence of LPLA2 at pH 7.4 or 5.5 (n = 3 or 6 independent experiments for t60 and t0, respectively). Data from PC:PG (90:10) liposomes, also presented in Fig.1, are added as a reference. Data are presented as mean±s.e.m..

Supplementary Figure 2

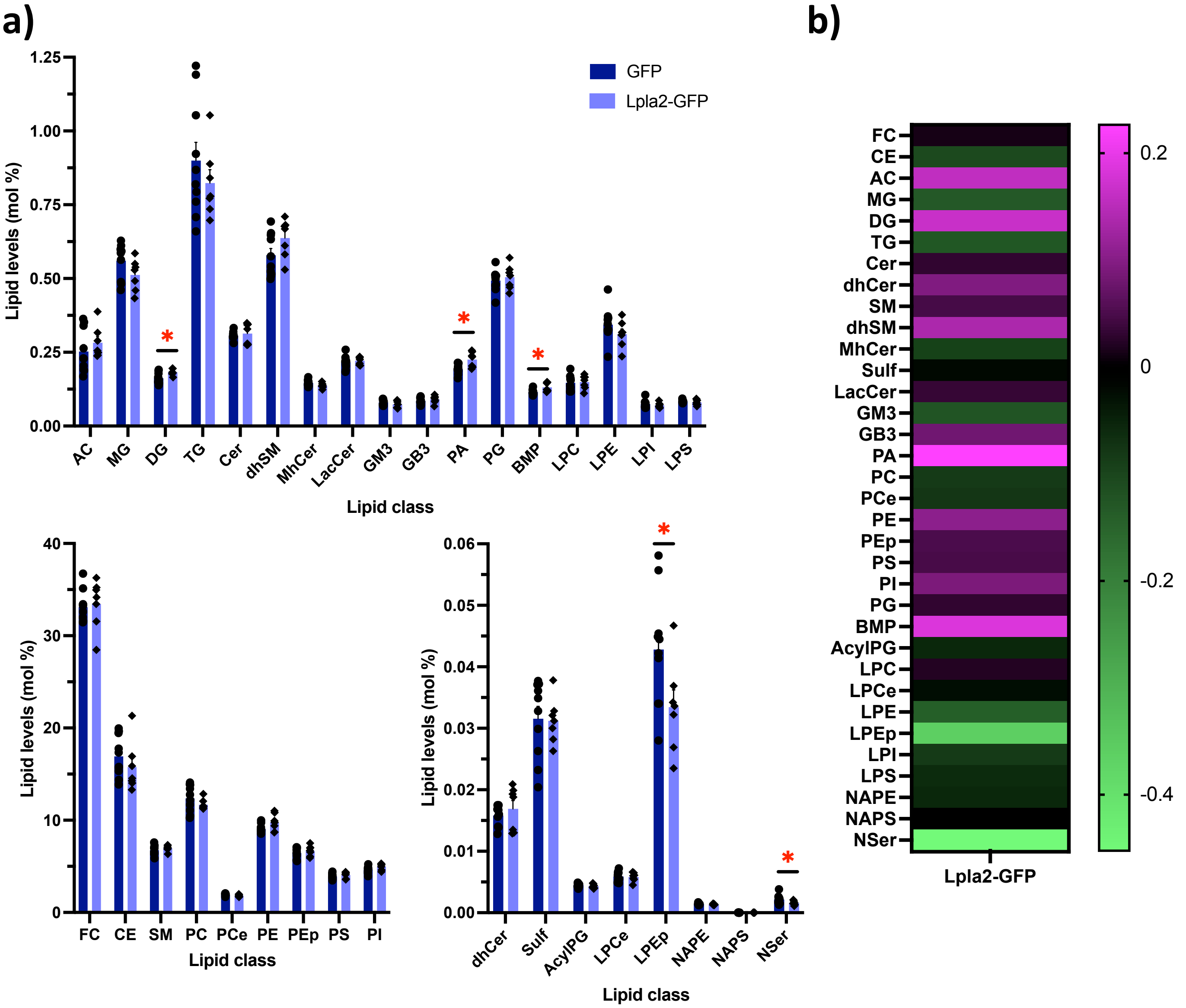

Supplementary Figure 2. Comparative lipid profiles of HeLa cells expressing Pla2-GFP *vs.* GFP. a) Total Lipid Composition. Dark blue bars: GFP (n = 10 transfections); light blue bars: Lpla2-GFP (n = 7 transfections).* Stands for $p < 0.05$ in multiple unpaired t-tests with Welch's correction. Data are presented as mean±s.e.m.. **b)** Heatmap representation of the effect of Pla2-GFP overexpression on lipid species. Log2 fold change values are represented in gradient color: magenta indicates an increase and green a decrease compared to cells expressing GFP only.

Supplementary Figure 3

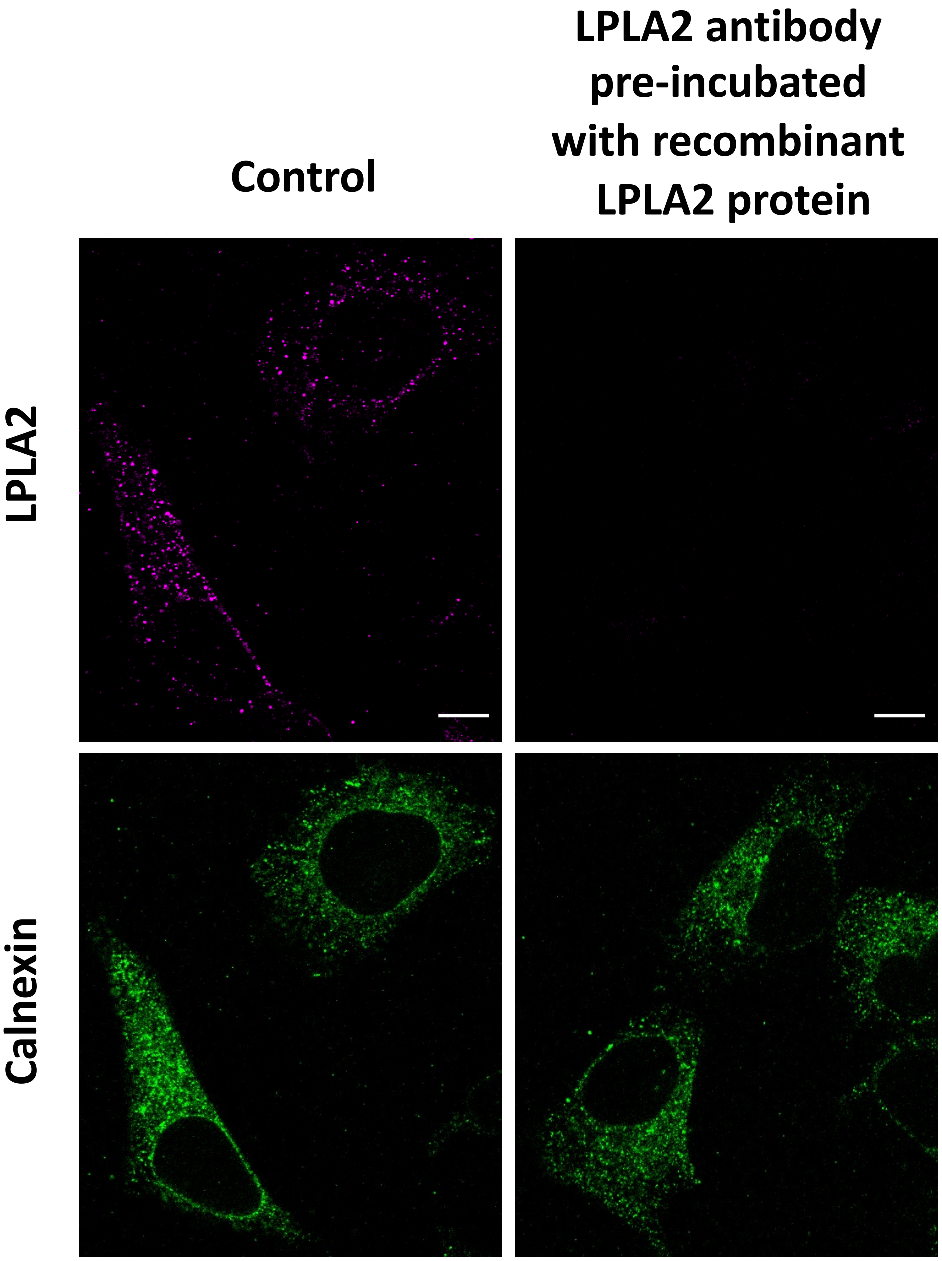

Supplementary Figure 3. Specificity of the LPLA2 antibody for immunocytochemistry. Representative section from a z-stack of HeLa cells immunostained for endogenous Calnexin (green) and LPLA2 (magenta) in control conditions (PBS, left) or with pre-incubation with the purified LPLA2 protein (right). Calnexin staining was used to localize the cells. Scale bar, 10 μm.

Supplementary Figure 4

a) b)

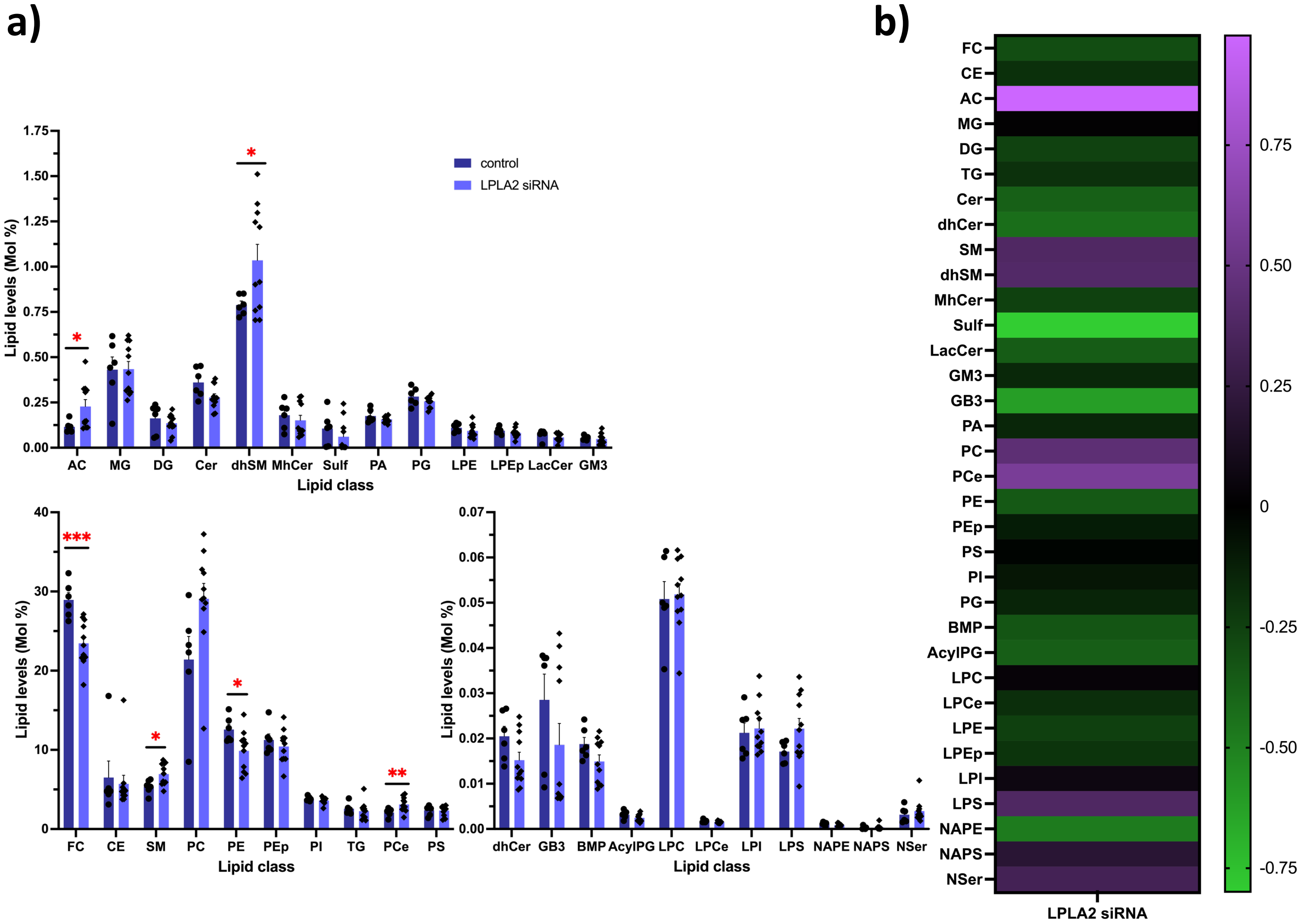

Supplementary Figure 4. Comparative lipid profiles of HeLa cells treated with a control siRNA or a siRNA targeting LPLA2. a) Total Lipid Composition. Dark blue bars: control siRNA (n = 6 treatments); light blue bars: LPLA2 siRNA (n = 11 treatments).*, ** and *** stand for $p < 0.05$; $p < 0.01$ and $p < 0.001$, respectively, in multiple unpaired t-tests with Welch's correction. Data are presented as mean±s.e.m.. **b)** Heatmap representation of the effect of a treatment with siRNAs targeting LPLA2 on lipid species. Log2 fold change values are represented in gradient color: magenta indicates an increase and green a decrease compared to cells treated with control siRNAs.

Supplementary Figure 5

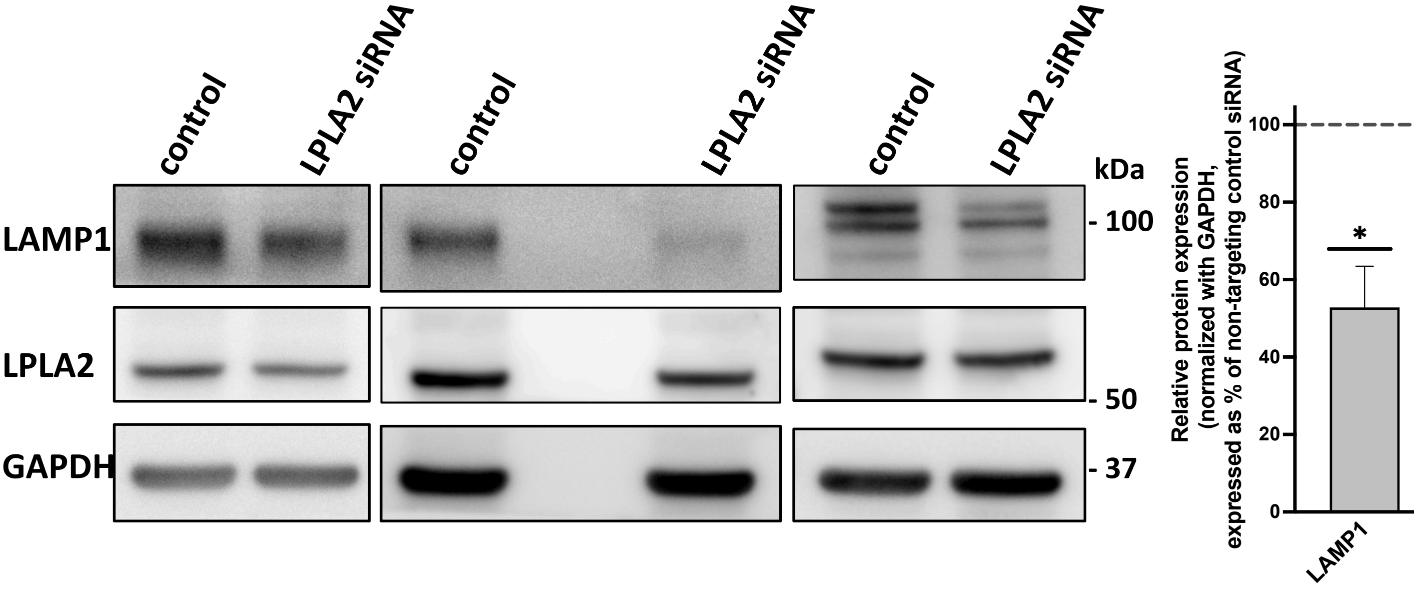

Supplementary Figure 5. Lamp1 levels are decreased in LPLA2 KD cells. Left, uncropped western blots of LAMP1, LPLA2 and GAPDH for HeLa cells treated with control or LPLA2 siRNA (3 independent experiments). GAPDH was used as an equal loading marker. Right, quantification of LAMP1 levels in LPLA2 KD cells (53±11%, n=3 experiments), $p<0.05$ in one sample t-test (theoretical mean 100%).
